# Prevalence of pre-gestational and gestational smoking and factors associated with smoking cessation during pregnancy, Brazil, 2011-2012

**DOI:** 10.1371/journal.pone.0217397

**Published:** 2019-05-24

**Authors:** Rosa Maria Soares Madeira Domingues, Valeska Carvalho Figueiredo, Maria do Carmo Leal

**Affiliations:** 1 Laboratório de Pesquisa Clínica em DST/Aids, Instituto Nacional de Infectologia Evandro Chagas, Fundação Oswaldo Cruz, Rio de Janeiro, Brasil; 2 Departamento de Epidemiologia e Métodos Quantitativos em Saúde, Escola Nacional de Saúde Pública Sérgio Arouca, Fundação Oswaldo Cruz, Rio de Janeiro, Brasil; University of South Florida, UNITED STATES

## Abstract

Gestational smoking is associated with various adverse maternal and fetal outcomes. Over the last three decades, despite considerable advances in tobacco control policy in Brazil, gestational smoking has caused a considerable number of fetal deaths and disabilities. The aim of this study is to estimate the prevalence of pre-gestational and gestational smoking and to identify the factors associated with smoking cessation up to the 20^th^ gestational week. Methods: “Birth in Brazil” was a nationwide hospital-based study conducted from February 2011 to October 2012. Smoking prevalence and smoking cessation during pregnancy was estimated through face-to-face interviews with postpartum women during hospitalization for birth care. We performed multivariate logistic regression to verify the factors associated with smoking cessation up to the 5th month of pregnancy. Results: prevalence of pre-gestational smoking of 16.1% (CI 95% 15.3%-16.9%); prevalence of smoking any time during pregnancy of 9.6% (CI 95% 9.0%-10.3%); and prevalence of smoking cessation up to the 5th month of pregnancy of 56.7% (CI 95% 54.0%-59.4%). The factors associated with smoking cessation were residence in the North, Northeast, and Central-West of Brazil, having received at least one prenatal consultation (OR 3.51 CI 95% 1.85–6.67), more years of schooling (15 or more vs less than 7 years of schooling OR 5.54 CI 95% 2.97–10.34), living with a partner (OR 1.35 CI 95% 1.01–1.79), no children prior to the index pregnancy (OR 2.77 CI 95% 2.13–3.61), and absence of alcohol use (1.74 CI 95% 1.39–2.18) or of suspected alcohol abuse (OR 1.62 CI 95% 1.07–2.45). Conclusion: The estimated smoking rate during pregnancy in Brazil is still high and is associated with factors of increased maternal social vulnerability, which may contribute to the increased occurrence of unfavorable perinatal outcomes.

## Introduction

Smoking during pregnancy is a major public health problem, associated with various adverse maternal and fetal outcomes [1]. Maternal smoking during pregnancy increases the risk of ectopic pregnancy, placenta previa, placental abruption, and miscarriage [2–4]. Studies have consistently identified an association between smoking during pregnancy and prematurity, intrauterine growth restriction, small for gestational age newborns, and cleft lip [2, 5–9]. A meta-analysis of 34 articles from around the world showed that smoking during pregnancy increases the risk of stillbirth by 47% and that the risk is dose-dependent [10].

Based on an extensive review including 295 studies that reported national data from 1985 to 2016, the global prevalence of smoking during pregnancy was estimated. There is considerable regional disparity due to the distinct social and cultural contexts: Europe has the highest prevalence (8.1%) and Africa the lowest (0.8%) [11]. Single marital status, low schooling, previous children, unplanned pregnancy, partners that smoke, early initiation of smoking, and alcohol abuse are associated with higher risk of continuing to smoke during pregnancy [12–14].

In Brazil, comprehensive tobacco control policies have been implemented over the past three decades and were intensified in 2005 with the ratification of World Health Organization Framework Convention on Tobacco Control (WHO FCTC) [15]. However, despite the considerable decline in prevalence overall and among women (25.3% in 1989 to 11.2 in 2013) [16–18], the number of maternal-fetal deaths in this period was devastating. A study based on available data from population surveys estimated that between 1989 and 2015, smoking during pregnancy caused 410,065 deaths due to low birth weight, 358,821 due to prematurity, and 25,070 due to placenta previa or placental abruption [19]. The authors found that tobacco control policies saved 345,403 lives ascribed to such causes, however all of these deaths could have been avoided through smoking cessation before and during pregnancy.

Importantly, since pregnant women wish to protect their infants’ health, pregnancy is a favorable time for smoking cessation [20, 21]. As with smoking itself, cessation also varies widely between countries. In a systematic review of 19 studies, the cessation rate varied from 4.0% to 69.7% for population-based studies and from 26.5% to 47.0% for hospital-based studies [22]. Factors directly related to cessation during pregnancy feature prenatal care, lower parity, presence of a partner, and higher socioeconomic status [13, 22–23]. One of the main factor inhibiting cessation is the level of dependence *vis a vis* the role of smoking in coping with pregnancy related types of stress. The decision of quitting smoking for health reasons is usually threatened by the relaxing effects of nicotine in a context of heavy dependence of this drug and a critical moment of transformation and stress such as pregnancy [22].

Article 14 of the WHO FCTC demands that states parties should offer adequate treatment for nicotine dependence [24], and the WHO recommends that all health professionals should offer social support and cessation counseling to all pregnant women seen at health services [25]. In Brazil, little is known about the policy’s impact on smoking cessation during pregnancy [26]. The aim of this study is to estimate the prevalence of pre-gestational smoking, the prevalence of smoking during pregnancy and factors associated with smoking cessation up to the 20^th^ gestational week.

## Methods

“Birth in Brazil” (NB) was a nationwide hospital-based study conducted from February 2011 to October 2012 that aimed to assess the conditions in prenatal, labor, and childbirth care in the country and the results of this care.

Calculation of the sample size was based on cesarean section as the outcome, estimated at 46.6%, with 5% significance to detect differences of 14% between types of services, study power of 95%, and design effect of 1.3.

The sample was selected in three stages. This first stage consisted of hospitals with 500 or more births per year, stratified by Brazil’s five major geographic regions, location (state capitals versus non-state capitals), and type of hospital (private, public, and mixed). The second stage was a calculation of the number of days that the interviewers needed to be in each maternity hospital to interview 90 puerperal women, considering a minimum of 7 days of research staff stay in each service so that women with deliveries on all days of the week could be included. The third stage consisted of selection of postpartum women. In hospitals with less than 90 deliveries per week, all women meeting the inclusion criteria were included sequentially until 90 interviews were reached. In hospitals with more than 90 deliveries per week, we conducted a random sample of all eligible women hospitalized during the 7 days during which the research team was in the hospital. In all, 23,894 postpartum women were interviewed in 266 hospitals located in 199 cities in all five regions of Brazil. For more information on the sampling design, see Vasconcellos et al. [27].

Inclusion criteria were: postpartum women that had given birth in hospital, with live birth as the outcome, independently of birth weight or gestational age, or stillbirth with birth weight greater than 500g or gestational age greater than 22 weeks.

The analysis used data obtained from an interview conducted with the mother while still in hospital, at least 6 hours after delivery, using an electronic questionnaire prepared specifically for this study. Data from the prenatal card, when available, were photographed digitally for subsequent extraction and digitization of data on an online platform. The entire process was conducted by a team of students and health professionals trained and supervised by the study’s central coordinating team. For more detailed information on the data collection, see do Carmo Leal et al [28].

To measure smoking, the mother was asked during the interview i) whether she had smoked before the pregnancy, ii) during the first 5 months of pregnancy, iii) after the fifth month of pregnancy, iv) whether she had smoked daily during pregnancy, and v) the number of cigarettes smoked per day during pregnancy. All variables, with the exception of the number of cigarettes smoked per day, were binary (yes or no). Based on this information, we estimated the prevalence rates for i) pre-gestational smoking, ii) smoking any time during the pregnancy, iii) smoking in the first 5 months of pregnancy, iv) smoking throughout the pregnancy (before and after the fifth month of pregnancy), v) daily smoking and mean number of cigarettes before and after the fifth month of pregnancy, vi) reduction (in frequency and/or amount) of smoking during pregnancy (comparing smoking frequency and amount in the first 5 months of pregnancy and after the fifth month of pregnancy), vii) smoking cessation throughout the pregnancy (women with pre-gestational smoking who reported not having smoked any time during the pregnancy) and viii) smoking cessation up to the 5^th^ month of pregnancy (women with pre-gestational smoking that reported not having smoked after the 5^th^ month of pregnancy).

To check for differences between pre-gestational and gestational smoking rates according to maternal characteristics and access to prenatal services, we used the chi-square test with significance set at 0.05. The maternal characteristics we analyzed were maternal region of residence (North, Northeast, South, Southeast and Central-West), maternal age (12 to 19, 20 to 34, 35 years and older), maternal schooling in years, self-reported skin color (white, black, brown, Asian-descendant, and indigenous, as measured in the Brazilian demographic census), conjugal situation (living versus not living with a partner), and paid work (yes versus no). Lifestyle factors included alcohol consumption, using the Tolerance Worry Eye-opener Annoyed Cut-down (TWEAK) scale with a score of 2 for suspected alcohol abuse [29]. Obstetric characteristics included parity (zero, 1 or 2, 3 or more previous births), history of previous negative perinatal outcomes (prematurity, low birth weight, stillbirth, and/or neonatal death), and whether the current pregnancy was planned or not (wanted to become pregnant now; wanted to become pregnant, but not now; did not want to become pregnant). As indicators of access to prenatal and birth care, we verified the proportion of women with at least one prenatal visit, the service where prenatal care was performed (public versus private), timing of first prenatal visit (1^st^, 2^nd^, or 3^rd^ gestational trimester), proportion of mothers with adequate number of prenatal visits, based on gestational age at the time of childbirth and a minimum of 6 visits for a low-risk term pregnancy (number of visits recommended by the Brazilian Ministry of Health at the time of the study) [30], type of birth care service (public, mixed or private) and hospital location (capital, non capital).

Considering that the harms from smoking during pregnancy for the outcome “birth weight” are more intense after the first trimester [31], we performed multivariate logistic regression to verify the factors associated with smoking cessation up to the 5^th^ month of pregnancy, the only data available in the survey on the timing of smoking cessation. As explanatory variables, the analysis included maternal characteristics and prenatal care (at least one prenatal visit). Variables with p < 0.20 in the univariate analysis were included in the multivariate model, and the final model included all the variables with p < 0.05. Asian-descendant and indigenous women were excluded from this analysis due to their small numbers (1.1% and 0.4%, respectively) which prevented the adjustment of the multivariate model.

The “Birth in Brazil” study used a complex sampling process, considered in all stages of the statistical analysis, with weighting, calibration, and design effect [27]. All the analyses used IBM SPSS Statistics for Windows, version 19.0 [32].

The study was approved by the Institutional Review Board of ENSP/Fiocruz under case review 92/2010. All precautions were taken to protect confidentiality. Before each interview, consent was obtained from each postpartum woman after reading the free and informed consent form.

## Results

Of the 23,894 women interviewed, 16.1% reported pre-gestational smoking, 9.6% reported smoking at some time in the pregnancy, and 6.8% reported smoking throughout the pregnancy. Of the pregnant women that reported smoking up to the 5^th^ month of pregnancy, 79.1% smoked daily, with a prevalence of 7.3% and mean daily consumption of 10.6 cigarettes. Among the pregnant women that smoked after the 5^th^ month of pregnancy, these figures were 80.8%, 5.8%, and 9.9, respectively. Approximately 65% of the women that reported smoking up to the 5^th^ month of pregnancy reduced their consumption of cigarettes during the pregnancy (frequency and/or amount), while 42.5% reported having quit smoking throughout the entire pregnancy (Table 1).

**Table 1:** Indicators for smoking during pregnancy. Brazil 2011–2012.

Indicator	(N = 23,894)	Proportion	CI 95%
	n		
Pre-gestational smoking	3,834	16.1	15.3–16.9
Smoking any time during pregnancy	2,296	9.6	9.0–10.3
Smoking throughout the pregnancy#	1,619	6.8	6.3–7.3
Smoking up to the 5^th^ month of pregnancy	2,201	9.2	8.6–9.9
Daily smoking up to the 5^th^ month of pregnancy	1,741	7.3	
Mean daily consumption (no. cigarettes)		10.6	10.0–11.1
Smoking after the 5^th^ month of pregnancy	1,714	7.2	6.7–7.7
Daily smoking after the 5^th^ month of pregnancy	1,382	5.8	
Mean daily consumption after the 5^th^ month of pregnancy		9.9	9.3–10.4
Reduction of smoking in the pregnancy (frequency and/or amount)	2,474	64.5	61.9–67.0
Smoking cessation during the pregnancy*(n = 3,834)	1,629	42.5	39.7–45.2
Smoking cessation up to the 5^th^ month of pregnancy**(n = 3,834)	2,171	56.7	54.0–59.4

# Smoking before and after the 5^th^ month of pregnancy

*Postpartum women with report of pre-gestational smoking that had not smoked any time during the pregnancy.

** Postpartum women with report of pre-gestational smoking that had not smoked after the fifth month of the pregnancy.

Higher prevalence rates for pre-gestational and gestational smoking were found in women living in the South and Southeast of Brazil, who received birth care in public and mixed hospitals, with black skin color, without paid work and not living with a partner. There was an inverse relationship between the likelihood of pre-gestational and gestational smoking and years of education, alcohol use, and parity, where the highest prevalence rates were seen in women with less than 8 years of schooling, with suspicion of alcohol abuse, and with more children. Higher prevalence rates for pre-gestational and gestational smoking were also seen in women with proportionally more negative outcomes in previous pregnancies and in those who had not wanted to become pregnant, and these differences were significant. No differences were observed according to maternal age or hospital location (state capitals versus non-state capitals) (Table 2).

**Table 2:** Pre-gestational and gestational smoking according to maternal characteristics. Brazil, 2011–2012.

Maternal characteristics	N = 23,894	Pre-gestational smoking (N = 3,830)			Gestational smoking (N = 2,295)		
	%*	%**	CI 95%	p value	%**	CI 95%	p value
**Region**							
North	9.6	13.7	11.6–16.2	<0.001	5.9	4.7–7.4	<0.001
Northeast	28.9	10.0	8.7–11.4		5.5	4.7–6.5	
Southeast	42.5	18.7	17.4–20.2		11.7	10.5–13.0	
South	12.5	23.3	21.2–25.5		15.1	13.5–17.0	
Central-West	6.5	15.1	13.1–17.4		9.0	7.4–10.8	
**Type of Hospital**							
Public	41.2	17.0	15.6–18.5	<0.001	10.8	9.7–12.0	<0.001
Mixed	44.0	18.1	17.0–19.3		10.9	9.9–11.9	
Private	14.8	7.3	6.5–8.2		2.6	2.0–3.3	
**Hospital location**							
Capital	37.3	17.0	15.5–18.6	0.104	9.1	7.9–10.5	0.334
Non capital	62.7	15.5	14.6–16.5		9.9	9.2–10.6	
**Age**							
12 to 19	19.1	16.1	14.2–18.2	0.854	9.1	8.0–10.3	0.554
20 to 34	70.4	16.0	15.0–16.9		9.7	9.0–10.5	
35 or more	10.5	16.6	14.8–18.5		10.0	8.5–11.7	
**Self-reported skin color**							
White	33.8	15.9	14.6–17.3	0.001	9.2	8.1–10.3	0.004
Black	8.6	20.5	18.1–23.1		12.8	10.8–15.0	
Mixed	56.1	15.5	14.5–16.5		9.4	8.7–10.2	
Asian-descendant	1.1	15.9	11.7–21.2		9.7	6.4–14.4	
Indigenous	0.4	12.4	6.0–23.9		6.3	2.6–14.6	
**Years of schooling**							
0–7	26.6	24.0	22.4–25.7	<0.001	16.9	15.5–18.4	<0.001
8–10	25.6	18.3	16.8–19.9		11.3	10.2–12.5	
11–14	39.0	11.4	10.2–12.7		5.4	4.7–6.1	
15 or more	8.9	6.4	5.3–7.8		1.5	1.0–2.3	
**Paid job**							
No	59.7	18.0	16.9–19.2	<0.001	11.2	10.3–12.1	<0.001
Yes	40.3	13.1	12.2–14.1		7.3	6.6–8.0	
**Conjugal situation**							
Living without partner	18.6	23.8	21.7–26.0	<0.001	16.4	14.8–18.1	<0.001
Living with partner	81.4	14.3	13.6–15.1		8.1	7.5–8.7	
**Alcohol** ***							
No use	86.0	12.5	11.8–13.4	<0.001	6.8	6.3–7.4	<0.001
No suspicion of alcohol abuse	4.0	23.4	19.9–27.3		15.4	12.8–18.6	
Suspicion of alcohol abuse	10.0	39.0	35.5–42.7		28.5	26.0–31.2	
**Parity**							
Primiparous	46.9	11.9	10.9–13.0	<0.001	5.6	5.0–6.2	<0.001
1or 2 previous births	42.7	17.5	16.5–18.6		11.3	10.5–12.3	
3 or more previous births	10.4	28.9	26.7–31.3		20.8	18.9–23.0	
Negative outcomes in previous pregnancies (n = 12,682)****							
No	55.8	18.5	17.3–19.7	<0.001	12.0	11.1–13.0	<0.001
Yes	44.2	24.2	22.2–26.4		17.4	15.6–19.3	
**Wanted pregnancy**							
Yes	44.6	12.9	11.9–14.1		7.3	6.6–8.0	<0.001
Yes, but not now	25.5	14.6	13.1–16.3		7.3	6.4–8.4	
No	29.9	21.8	20.4–23.4		15.0	13.8–16.4	

* Column proportion

** proportion of line

***using the TWEAK scale with a score of 2 for suspected alcohol abuse

****women with previous births

Compared to women that did not report pre-gestational and gestational smoking, smokers showed lower coverage of prenatal care, more prenatal care in the public health system, later initiation of prenatal care, and lower proportion of adequate number of visits, and all these differences were significant (Table 3).

**Table 3:** Prenatal care during current pregnancy according to pre-gestational and gestational smoking. Brazil, 2011–2012.

Prenatal care	Pre-gestational smoking					Gestational smoking				
	No (N = 20,052)		Yes (N = 3,834)		p value	No (N = 21,598)		Yes (N = 2,296)		p value
	%	CI 95%	%	CI 95%		%	CI 95%	%	CI 95%	
**Prenatal care**					<0.001					<0.001
Yes	99.1	98.8–99.3	97.2	96.2–98.0		99.1	98.9–99.3	95.8	94.2–97.0	
No	0.9	0.7–1.2	2.8	2.0–3.8		0.9	0.7–1.1	4.2	3.0–5.8	
Type of service*					<0.001					<0.001
Public	68.4	66.7–70.1	83.3	81.5–84.9		68.9	67.2–70.5	89.3	87.4–90.9	
Private	31.6	29.9–33.3	16.7	15.1–18.5		31.1	29.5–32.8	10.7	9.1–12.6	
Timing of first prenatal visit *					<0.001					<0.001
1^st^ gestational trimester	61.9	60.3–63.4	53.5	51.1–55.8		61.6	60.2–63.1	49.7	47.4–52.1	
2^nd^ gestational trimester	34.9	33.5–36.3	40.7	38.5–43.0		35.1	33.7–36.4	42.9	40.6–45.3	
3^rd^ gestational trimester	3.3	2.9–3.7	5.8	4.6–7.3		3.3	2.9–3.7	7.3	6.1–8.9	
**Adequate number of prenatal visits** **					<0.001					<0.001
No	23.1	21.9–24.4	32.8	30.6–35.1		23.4	22.2–24.6	37.0	34.3–39.8	
Yes	76.9	75.6–78.1	67.2	64.9–69.4		76.6	75.4–77.8	63.0	60.2–65.7	

*Women who attended antenatal care

**Adequate number of prenatal visits: based on gestational age at the time of childbirth and a minimum of 6 visits for a low-risk term pregnancy.

Prevalence of smoking cessation up to the 5^th^ month of pregnancy was 56.7% (95% CI 54.0–59.4). In the univariate analysis, all factors tested, except self-reported skin color and paid job, were associated with smoking cessation (Table 4).

**Table 4:** Univariate analysis of factors associated with smoking cessation up to 20 weeks of gestation. Brazil, 2011–2012.

Maternal characteristics	Smoking cessation up to the 5^th^ month of pregnancy (N = 3,842)			
	%	OR	CI 95%	p value
**Region (n = 3,842)**				
Southeast	53.1	1		< 0.001
North	75.1	3.01	1.92–4.70	
Northeast	62.6	1.55	1.09–2.19	
South	53.4	1.06	0.82–1.35	
Central-West	54.3	1.14	0.78–1.66	
**Hospital location (n = 3,842)**				
Capital	61.4	1.38	1.07–1.79	0.004
Non capital	53.7	1		
**Age (n = 3,839)**				
12 to 19	65.3	1.86	1.23–2.81	0.009
20 to 34	54.9	1.15	0.86–1.53	
35 or more	53.1	1		
**Years of schooling (n = 3,822)**				
15 or more	86.0	7.66	3.90–15.04	<0.001
11 to 14	68.1	2.20	1.74–2.78	
8 to 10	55.6	1.29	1.01–1.65	
0 to 7	47.2	1		
Self-reported skin color (n = 3,785)*				
White	59.6	1.42	0.94–2.15	0.219
Black	49.9	1		
Mixed	56.0	1.30	0.83–2.01	
**Conjugal situation (n = 3,835)**				
Living with patner	58.4	1.30	0.99–1.71	0.060
Living without partner	52.4	1		
**Paid job (n = 3,837)**				
Yes	58.4	1.03	0.86–1.24	0.739
No	55.9	1		
**Alcohol (n = 3,648)**				
No	60.0	1.77	1.36–2.30	<0.001
No suspicion of alcohol abuse	58.3	1.57	1.01–2.45	
Suspicion of alcohol abuse	46.4	1		
**Parity (n = 3,842)**				
Primiparous	72.4	3.38	2.60–4.39	<0.001
1or 2 previous births	50.9	1.28	0.98–1.67	
3 or more previous births	42.1	1		
**Wanted pregnancy (n = 3,804)**				
Yes	59.1	1.56	1.24–1.98	<0.001
Yes, but not now	66.9	2.09	1.62–2.70	
No	48.7	1		
Negative outcomes in previous pregnancies** **(n = 2,503)**				
No	59.0	1.61	1.29–2.02	<0.001
Yes	45.6	1		
**Prenatal care (n = 3,835)**				
No	21.2	1		
Yes	57.7	5.03	2.88–8.78	<0.001

*Asian-descendant and indigenous women were excluded from this analysis

** only women with previous pregnancies

In the multivariate analysis (Table 5), the factors associated with smoking cessation were residence in the North, Northeast, and Central-West of Brazil, having received at least one prenatal consultation, more years of schooling, living with a partner, no children prior to the index pregnancy, and absence of alcohol use or of suspected alcohol abuse. Women who wanted to become pregnant, but not now, also showed higher odds of smoking cessation up to the 5^th^ month of gestation, while women who had wanted to become pregnant showed higher cessation rates, but the difference was not statistically significant. As with the prevalence of pre-gestational and gestational smoking, there was a relationship between the likelihood of smoking cessation and years of schooling: women with complete university education had 5.5 higher odds of quitting smoking when compared to women with incomplete primary schooling. Hospital location, age and negative outcomes were not kept in the final model as they had a p-value >0.05 after adjusting for other variables.

**Table 5:** Multivariate analysis of factors associated with smoking cessation up to 20 weeks of gestation, Brazil 2011–2012.

Maternal characteristics	Smoking cessation up to the 5^th^ month of pregnancy (N = 3,842)			
	%	OR adj*	CI 95%	p value
**Region (n = 3,842)**				
Southeast	53.1	1		<0.001
North	75.1	3.98	2.50–6.33	
Northeast	62.6	2.17	1.48–3.18	
South	53.4	1.12	0.86–1.46	
Central-West	54.3	1.48	1.01–2.17	
**Years of schooling (n = 3,822)**				
15 or more	86.0	5.54	2.97–10.34	<0.001
11 to 14	68.1	2.21	1.68–2.91	
8 to 10	55.6	1.31	0.98–1.74	
0 to 7	47.2	1		
**Conjugal situation (n = 3,835)**				
Living with patner	58.4	1.35	1.01–1.79	0.042
Living without partner	52.4	1		
**Alcohol (n = 3,648)**				
No	60.0	1.74	1.39–2.18	<0.001
No suspicion of alcohol abuse	58.3	1.62	1.07–2.45	
Suspicion of alcohol abuse	46.4	1		
**Parity (n = 3,842)**				
Primiparous	72.4	2.77	2.13–3.61	<0.001
1or 2 previous births	50.9	1.18	0.91–1.54	
3 or more previous births	42.1	1		
**Wanted pregnancy (n = 3,804)**				
Yes	59.1	1.07	0.85–1.34	0.017
Yes, but not now	66.9	1.47	1.13–1.91	
No	48.7	1		
**Prenatal care (n = 3,835)**				
No	21.2	1		<0.001
Yes	57.7	3.51	1.85–6.67	

* Final model adjusted for region, schooling, conjugal situation, parity, wanted pregnancy, and prenatal care.

## Discussion

“Birth in Brazil” is the first nationwide study to assess prenatal, labor, and childbirth care in the country. Its sound methodology with clear inclusion criteria of hospitals and women, adequate sample size, appropriate sampling and data collection procedures to avoid selection and information bias, as well as appropriate data analysis, has provided information around many aspects of pregnancy and childbirth care. Therefore, our results represent the best national estimates of prevalence rates for smoking and smoking cessation during pregnancy and the associated factors. In a country with comprehensive measures of tobacco control and downward trend in smoking prevalence, little was known about smoking during pregnancy at the national level. By filling this gap, this study has provided the necessary knowledge for the induction of specific measures aimed at this population group and might be used as baseline for future evaluation studies.

Our results are consistent with those of previous studies on this topic. The regional and socioeconomic profile of pre-gestational smoking is similar to that of Brazilian women 18 years and older in general: the South and Southeast regions, and more vulnerable groups such as blacks and those with less schooling, with higher smoking rates [16, 17].

Still, smoking prevalence in the current study (16.5%) was higher than in Brazil’s adult female population in general (11.2%) [17]. There are three possible explanations for this finding. The first is the study period. The Birth in Brazil study was done in 2011–2012, with most of the interviews performed in 2011, while the National Health Survey [17] was conducted in 2013. Since there is a downward trend in smoking prevalence in Brazil [16], part of the difference may be due to the year when the interviews were held. The second explanation is the way pre-gestational smoking was measured in the Birth in Brazil study, without specifying the target period. Some women may have reported pre-gestational smoking any time in life, while the National Health Survey measured current smoking; in other words, the Birth in Brazil study may have overestimated the prevalence. Note that prevalence of past smoking by women was 14.1% in the National Health Survey [17]. Finally, differences in the two study samples in terms of schooling, skin color, and age could partly explain the higher prevalence of smoking by women included in Birth in Brazil, with relatively higher social vulnerability. An example is prevalence of smoking by age bracket. The National Health Survey [17] included women 18 to 60 years or older, while Birth in Brazil included females 10 to 49 years of age. In the National Health Survey, women 18 to 24 years of age showed the lowest smoking rate, while Birth in Brazil did not show any differences according to age bracket.

The estimated prevalence of maternal smoking throughout pregnancy in Birth in Brazil, of 7.2%, is lower than in most European and American countries. In a routine prenatal data assessment including 23 European countries, smoking prevalence in the third trimester varied from less than 5% in Lithuania and Sweden to 19% in Scotland. Of the 23 countries, only four recorded rates similar to or lower than Brazil [33]. However, the rates in Brazil varied greatly between the major geographic regions, with the South and Southeast showing rates close to or as high as most of the European countries.

Comparing Brazil to other countries of the Americas, the estimated prevalence of smoking in late pregnancy was similar to that in the United States (7.6% in 2016) [13]. According to recent studies, Argentina (16.1%), Uruguay (26.7%), and Chile (11.3%) showed higher rates than the average for Latin America as a whole and similar to the South and Southeast of Brazil [34, 35]. The rate in Uruguay (26.7%) was far higher [21, 36].

Local studies in Brazil that estimated the prevalence of maternal smoking any time in pregnancy, all found higher rates than in the Birth in Brazil study at the corresponding regions. Kale et al. (2015) studied pregnant women in two maternity hospitals in the Southeast of Brazil and found smoking prevalence rates of 24.8% and 17.9% [23], compared to 11.7% in the Birth in Brazil study. In birth cohorts in a city in the South of Brazil, 22% of mothers were smokers in 2007 and 18% in 2013 [37], higher than the prevalence of 15.1% estimated by the Birth in Brazil study. In a cross-sectional study of 330 pregnant women treated in primary care in the Central-West of Brazil, 37.1% had smoked during pregnancy [38], four times more than in the Birth in Brazil study. One possible explanation for the difference is that the Birth in Brazil study included pregnant women seen in private maternity hospitals, who show considerably lower smoking rates than those seen in the public system [37].

Smoking cessation varies widely between continents and even within the same country. This variation can be explained by methodological differences, the study population’s characteristics, and cultural contexts and suggests the importance of some caution when making comparisons. When compared only to hospital-based studies, smoking cessation in the Birth in Brazil study (42.5%) was similar to that reported by Tong et al. in the United States [39]. Smoking cessation in our study was higher than in pregnant women in Uruguay and Argentina [21] and lower than in Canada [40]. When compared to population-based and hospital-based studies [22], smoking cessation in pregnant women in Birth in Brazil was similar to that in pregnant women in Sweden (47.0%) and higher than in studies in the United States (26.7%-37.5%), Australia (27.0%), New Zealand (26.8%), and Spain (26.5%).

Based on studies in Brazil, the smoking cessation rates up to the second trimester in two public maternity hospitals in Rio de Janeiro were 22.2% and 35.0% [23]; in three birth cohorts in the South of Brazil from 2007, 2010, and 2013, the rates were 18.0%, 21.1%, and 17.6% [37], respectively; and a study that included 267 postpartum women in a university hospital found 25.5% [41]. Thus, in all these studies, smoking cessation during pregnancy was lower than in the Birth in Brazil study. There are two possible explanations for this finding: 1) the studies cited in this case are concentrated in the South and Southeast of Brazil, where women’s smoking rates are higher, and where the odds of cessation are lower when compared to other regions of the country and 2) the Birth in Brazil study included women that had given birth in private maternity hospitals, with higher mean socioeconomic status and higher odds of cessation, only present in the cohort studies in the South [37].

Persistence of maternal smoking during pregnancy was heavily associated with low schooling, as identified in various studies conducted in Brazil and elsewhere in the world [22, 37, 42, 43]. Smoking is concentrated proportionally in low-income populations and is an important cause of health inequality [44]. In Brazil, women with up to 7 years of schooling have 70% higher prevalence of smoking than those with 8 years of education or more [45]. The relationship between smoking and inequality, including in pregnancy, is explained by various economic, social, and contextual factors. Socially underprivileged women tend to begin smoking earlier, have higher nicotine dependence levels, are more influenced by parents and friends that smoke, are more exposed to environmental smoke in the workplace and community, are less aware of the risks associated with smoking, and show higher rates of psychiatric comorbidities [46]. Various authors have reported that negative social conditions lead to stress and inability to cope with adversities. In this context, smoking tends to serve as a form of relaxation and mood regulation, especially in women, since the dependence can be reinforced more by the sensory and social context that by the nicotine itself [47, 48].

Another finding that refers to the stress paradigm is the strong relationship between the persistence of maternal smoking and not living with a partner, unwanted pregnancy, and number of children [12, 22]. Single motherhood, a factor closely related to unplanned pregnancy, usually involves less social support, which hinders cessation and use of prenatal care [40]. The number of children reinforces the theory that women smoke to compensate for moments of stress and pressure. Combined with low socioeconomic status, this underscores the importance of approaches to cessation that consider pregnant women’s social context in order to increase the effectiveness of interventions.

The association between smoking and alcohol abuse and the lower smoking cessation rates in women with abusive use are well established in the international and Brazilian literature [12, 38, 42]. They often involve the existence of psychiatric co-morbidities such as depression, expressing economic disadvantages and hindering cessation and access to prenatal care. These women thus have cumulative risks of exposure to both risk factors, increasing the risks of negative perinatal outcomes [49].

In addition to the above-mentioned social determinants, women in situations of vulnerability generally enjoy less access to health services [23, 42, 50]. Added to a favorable context for access, women with greater adherence to healthy practices are known to be more likely to seek prenatal care and thus show higher prenatal coverage. In this study, the beneficial effect of prenatal care was maintained even after adjusting for the social and obstetric variables. Women with greater social vulnerability and less access to adequate antenatal care, who showed the highest prevalence rates of pre-gestational smoking, were also the ones with the lowest smoking cessation rates up to the fifth month of pregnancy, highlighting the inequality in exposure to smoking during pregnancy.

This study had some limitations. The “Birth in Brazil” study was conducted in hospitals with more than 500 births per year, and the results cannot be extrapolated to women that gave birth at home, on public byways, or in smaller hospitals.

A second limitation is that the Birth in Brazil study did not include women that evolved with miscarriage. Since smoking during pregnancy causes early fetal losses, the non-inclusion of women hospitalized for miscarriage may have led to underestimation of smoking before and during pregnancy.

A third limitation is the fact that smoking was self-reported. There is widespread public awareness of the harms of smoking for the newborn and a known tendency for pregnant women to underreport smoking and over report cessation. However, diagnoses of smoking based on self-report compared to diagnoses by nicotine levels have demonstrated high sensitivity and specificity and a difference of only approximately 2% in the final prevalence estimates [51, 52]. Thus, although the smoking rates may be underestimated and the cessation rates overestimated, the differences are probably small, and there is no reason to infer that this trend differs according to the subgroups or that the profile of sociodemographic and gestational variables is affected.

As discussed above, pre-gestational smoking was measured without specifying a time period, and women may have reported smoking any time in life and not only in the moment prior to the index pregnancy, which may have overestimated the prevalence of pre-gestational smoking and cessation of smoking during pregnancy.

Finally, smoking during pregnancy was measured at only two time points, before and after the 5^th^ month of pregnancy, which is not the most appropriate, considering the results of recent studies indicating higher risk of negative perinatal outcomes after the first trimester. Besides, this measurement did not allow adequately estimating cessation after the 5^th^ month of pregnancy, since women that reported smoking after the 5^th^ month might have quit smoking before the end of the pregnancy.

## Conclusion

The estimated smoking rate during pregnancy in Brazil is still high and is associated with factors of increased maternal social vulnerability, which may contribute to the increased occurrence of unfavorable perinatal outcomes. The lower coverage of prenatal care in these women and the less adequate care may be contributing to lower smoking cessation rates in this group.

The study’s results show that Brazil should expand the scope of tobacco control measures and develop specific strategies integrated with prenatal care, aimed at early smoking cessation during pregnancy, aligned with World Health Organization guidelines, targeted to the most vulnerable population, which has the highest smoking prevalence rates.
